# 

**Published:** 1890-08

**Authors:** 


### CONTENTS.

COMMUNICATIONS.

Hereditary Character of Deformities ........ 369
Evolution in Dentistry ........ 375
The Value of Illustration in the Lecture Room ........ 379
Oil of Birch and Oil of Wintergreen ........ 381

PROCEEDINGS AND PAPERS.

Thirty-Fifth Annual Meeting of the Michigan State Dental Association ........ 386
Dental Ethics ........ 386
Treatment of Second Stage of Alveolar Abscess ........ 392
The Dental Protective Association ........ 398
Missouri State Dental Association ........ 405

COMMENCEMENTS.

University of Pennsylvania—Dental Department ........ 408
College of Dental Surgery of the University of Michigan ........ 409
Northwestern University—Dental Department ........ 409
Boston Dental College ........ 410
Harvard University—Dental Department ........ 410

CORRESPONDENCE.

Atlanta, Georgia ........ 411
Battle Creek, Michigan ........ 412

EDITORIAL.

Southern Dental Association ........ 417
Obituary ........ 419
In Memoriam ........ 420

